# Waterpipe (narghile) smoking among medical and non-medical university students in Turkey

**DOI:** 10.3109/03009734.2010.487164

**Published:** 2010-07-19

**Authors:** Serpil Poyrazoğlu, Şule Şarli, Zeliha Gencer, Osman Günay

**Affiliations:** Erciyes University Medical Faculty, Department of Public Health, KayseriTurkey

**Keywords:** Prevalence rate, socio-demographic factors, university students, waterpipe smoking

## Abstract

**Objectives:**

This investigation was performed in order to determine the prevalence rate of waterpipe smoking in students of Erciyes University and the effects of some socio-demographic factors.

**Methods:**

A total of 645 students who study the first three grades of the medical faculty and the engineering faculty of Erciyes University were enrolled in the study. A questionnaire including 48 questions was applied. Chi-square test and logistic regression method were performed for the statistical analyses.

**Results:**

The total prevalence rate of waterpipe smoking was found to be 32.7%. The prevalence rate of waterpipe smoking was 28.6% in the medical and 37.5% in the non-medical students. It was determined that 41.6% of the males and 20.2% of the females currently smoke waterpipe. Gender, cigarette smoking, and the presence of waterpipe smokers among family members and friends have significant effects on the prevalence of waterpipe smoking. Residence and economical status of the family and with whom the students live have no significant effect on the prevalence rate.

**Conclusions:**

Approximately one-third of the students currently smoke waterpipe. Smoking of both cigarette and waterpipe was frequently found. The measures against all tobacco products should be combined.

## Introduction

Tobacco use, one of the most important reasons for preventable mortalities, causes approximately 5 million deaths per year. This rate is estimated to increase to 10 million in 20–30 years. Of the deaths due to tobacco use, 70% are in the developing countries, and these countries are the ones in which problems due to epidemic tobacco use are mostly seen (1–3).

The most common form of tobacco use is cigarette smoking. The World Health Organization (WHO) estimates the number of smoking individuals as 1.1 billion, worldwide. A total of 700 million male smokers and 100 million female smokers are living in developing countries. In other words, 47% of the males living in developing countries and 7% of the females are smokers. Together with the marketing initiatives of the tobacco industry, and parallel to the constant increase of the population, tobacco use is also steadily increasing (1–3).

There are other ways of tobacco use apart from cigarettes; one of them is waterpipe use. Waterpipe is a tray connected to a bottle half full of water by way of a metal tube. The smokers inhale the smoke through a hose connected to the metal tube. The main part of the waterpipe is the tobacco called ‘tumbeki’. Tumbeki is usually wet, smelly, and sugary and is used by heating it above a piece of charcoal (4,5).

The waterpipe has a past of approximately 400 years and is a method preferred by the elderly. Waterpipe use has decreased substantially in the last century, but it has been spreading, especially among young people after the 1980s. This increase can be explained by the worldwide campaign against cigarette smoking (3,6).

There are 100 million daily waterpipe users worldwide. It is common on the Arabian Peninsula, in Turkey, India, Pakistan, Bangladesh, and some parts of China. In some places it is even more common than cigarette smoking (6). During the last years, a spread is noticed towards Europe and North America (6).

Nowadays, a kind of waterpipe tobacco called ‘bahri’ or ‘Arabian tumbeki’, brought from Egypt, is quite popular, especially among young people. These kinds of tumbekis are produced with sharp herbal or fruit aromas such as apple, mint, apricot, strawberry, and banana (7).

Substantial amounts of smoke can be inhaled during use of waterpipe. One waterpipe smoking period lasts for 30–60 minutes and contains approximately over 100 inhalations, each with an approximate volume of 500 mL. One cigarette produces approximately 500–600 mL of smoke, whereas one waterpipe smoking period produces 50,000 mL of smoke. Waterpipe smoke contains many of the same toxicants as cigarette smoke. Some of them are carbon monoxide in the respiratory air of waterpipe users and nicotine in their blood. The nicotine amount in the blood of daily waterpipe users is at the same level as those smoking 10 cigarettes a day (4).

Though there is more need for epidemiologic investigations, it is known that waterpipe use is related to important problems such as malignancies, cardiovascular system diseases, and nicotine addiction (4). One of the other probable health issues waterpipe use may cause is the risk of spreading infectious diseases (7).

Some factors such as the belief that waterpipe smoking is less harmful compared to cigarette smoking, its being easily attainable, and the cheap cost are held responsible for its spread, not only in Arabian countries but worldwide in all age groups (6).

No study was found investigating the waterpipe use in Turkey among university students.

This study aimed to investigate the prevalence of waterpipe use among university students and the effect of various socio-demographic and educational factors upon waterpipe smoking.

## Material and methods

The ethics committee of Erciyes University Medical Faculty approved this study and the directorate of Erciyes University gave permission.

Erciyes University is a state university in Kayseri, which is a province in the central part of Turkey. The data of the study were collected in the 2008–2009 educational period. The students who studied at the first three grades of the medical faculty and the engineering faculty of Erciyes University were planned to be included into the study. It was determined that there were 455 students at the medical faculty and 1411 students at the engineering faculty. It was thought to include a similar number of students from each faculty. For this reason, all of the 455 students at the medical faculty and 448 students who were studying three programmes at the engineering faculty were planned to be included.

An anonymous questionnaire which was prepared by the investigators and contained 48 questions was administered. Fifteen of the questions were related to socio-demographic characteristics, 22 were related to waterpipe smoking condition, 5 were related to the use of other tobacco products, and 6 were related to the students' opinions about waterpipe smoking. The students in the study group were visited at their class-rooms and were informed about the purpose of the study both verbally and in writing. Then the questionnaire was given. The questionnaire was completed by the students under the supervision of the investigators and then taken back. The students who were absent during the visit were not included in the study. None of the students who were in the class-rooms during the visit refused to answer the questionnaire. A total of 360 students from the medical faculty and 291 students from the engineering faculty answered the questionnaire. Six questionnaires were excluded because of incomplete answers. So a total of 645 (71.4%) questionnaires were evaluated.

Economic levels of the families were evaluated in three categories as high, moderate, and poor according to the reports of the students. In the evaluation of residence of the families, the provincial centres were accepted as ‘urban’, and other places were accepted as ‘rural’. Living arrangements of the students were evaluated in two categories as ‘with the family’ and ‘separate from the family’.

Chi-square test and logistic regression were applied for statistical analyses. In all statistical analyses, *P*-values less than 0.05 were accepted as statistically significant. In the logistic regression analysis, current waterpipe smoking condition was taken as dependent variable. All the independent variables in the logistic regression analysis were as follows:

- Gender: 1) Female (reference); 2) Male
- Faculty: 1) Medical (reference); 2) Engineering
- Grade: 1) I (reference); 2) II; 3) III
- Economic level of the family: 1) Poor (reference); 2) Moderate; 3) Good
- Residence of the family: 1) Rural (reference); 2) Urban
- Living arrangements: 1) With the family (reference); 2) Separate from the family
- Cigarette smoking: 1) Non-smoker (reference); 2) Smoker
- Waterpipe smoker among family members: 1) No (reference); 2) Yes
- Waterpipe smoker among friends: 1) No (reference); 2) Yes

## Results

The socio-demographic characteristics of the study group are given in Table I.

**Table I.** Socio-demographic characteristics of the study group.

Characteristics	Groups	*n*	%
Age (years) (mean ± SD)		20.3 ± 1.7	
Gender	Female	272	42.2
	Male	373	57.8
Faculty	Medical	357	55.3
	Engineering	288	44.7
Grade	I	290	45.0
	II	170	26.4
	III	185	28.7
Residence of the family	Rural	204	31.6
	Urban	441	68.4
Living arrangements	With the family	211	32.7
	Separate from the family	434	67.3
Economic level of the family	Poor	27	4.2
	Moderate	374	58.0
	Good	244	37.8
Total		645	100.0

Of the students in the study group 45.1% stated that they had experienced waterpipe. It was found out that 7.2% of the students who had experienced waterpipe had done so before 15 years of age, 41.3% between 15 and 17 years of age, and 47.7% after 17 years of age. Of the students who experienced waterpipe 92.4% said that they smoked waterpipe at a café for the first time.

The total prevalence rate of current waterpipe smokers was found to be 32.7%. The effects of various independent variables on the prevalence rate of waterpipe smoking were investigated, and odds ratios were calculated. Additionally, logistic regression analysis was performed in order to adjust the independent effects of these variables. Unadjusted and adjusted odds ratios are given in Table II.

**Table II.** The impacts of various factors on the prevalence rate of waterpipe smoking.

		Waterpipe smokers			
Characteristics	*n*	*n*	%	Unadjusted OR (95% CI)	Adjusted OR (95% CI)
Gender					
Female	272	55	20.2	1.00	1.00
Male	373	155	41.6	2.82 (1.96–4.04)^a^	2.22 (1.45–3.40)^a^
Faculty					
Medical	357	102	28.6	1.00	1.00
Engineering	288	108	37.5	1.50 (1.08–2.09)^a^	1.19 (0.80–1.76)
Grade					
I	290	88	30.3	1.00	1.00
II	170	52	30.6	1.01 (0.67–1.53)	1.15 (0.72–1.84)
III	185	70	37.8	1.40 (0.95–2.06)	1.35 (0.79–2.30)
Economic level of the family					
Poor	27	9	33.3	1.00	1.00
Moderate	374	11	29.7	0.84 (0.37–1.94)	1.45 (0.96–2.19)
Good	244	90	36.9	1.17 (0.50–2.71)	1.55 (0.55–4.36)
Residence of the family					
Rural	204	62	30.4	1.00	1.00
Urban	441	148	33.6	1.16 (0.81–1.65)	1.09 (0.70–1.69)
Living arrangements					
With the family	210	66	31.4	1.00	1.00
Separate from the family	433	144	33.3	1.09 (0.76–1.55)	1.31 (0.85–2.01)
Cigarette smoking					
Non-smoker	567	147	25.9	1.00	1.00
Smoker	78	63	80.8	12.00 (6.63–21.73)^a^	9.12 (4.76–17.46)^a^
Waterpipe smoker among family members					
No	576	163	28.3	1.00	1.00
Yes	69	47	68.1	5.41 (3.16–9.27)^a^	5.37 (2.93–9.86)^a^
Waterpipe smoker among friends					
No	391	82	21.0	1.00	1.00
Yes	254	128	50.4	3.83 (2.71–5.41)^a^	2.96 (1.98–4.43)^a^
Total	645	210	32.7		

^a^*P* < 0.05.

As shown in Table II, the gender and cigarette smoking condition of the students, and the presence of waterpipe smokers among family members and friends were found to have significant effects on the prevalence rate of waterpipe smoking of the students. It was found that male gender, cigarette smoking, presence of any waterpipe smoker among family members and friends significantly affect the probability of waterpipe smoking of the students. In the univariable analysis, the prevalence rate of waterpipe smoking among the non-medical students was significantly higher than among the medical students; however, in the logistic analysis, there was found no significant effect of the faculties on waterpipe smoking. On the other hand, economic condition and residence area of the family, and living arrangement of the student were found to have no significant effect on waterpipe smoking. Various characteristics of the current waterpipe smokers are given in Table III.

**Table III.** Distribution of waterpipe smokers according to various characteristics.

Characteristics	Groups	*n*	%
Age at the beginning of smoking waterpipe (years) (mean ± SD)		17.4 ± 2.2	
Frequency	Every day	1	0.5
	Once in 2–7 days	39	18.5
	Less than weekly	170	81.0
Place to smoke	Café	163	77.6
	Home	18	8.6
	Other places	29	13.8
Feeling hooked	Yes	9	4.3
	No	191	91.0
	Undecided	10	4.8
Smoking together with others	Alone	11	5.2
	With friends	192	91.4
	With family members	7	3.3
Share the waterpipe with others	Yes	182	86.7
	No	28	13.3
Suffer harm from waterpipe	Yes	22	10.5
	No	188	89.5
Reason for waterpipe smoking	Enjoyment	152	72.4
	Friend's demand	26	12.4
	Habit	1	0.5
	Others	31	14.8
Thinking of stopping smoking	Yes	44	21.0
	No	131	62.4
	Undecided	35	16.7
Total		210	100.0

The mean age at the beginning of waterpipe smoking was found to be 17.4 ± 2.2 years. Only one student stated that he smoked waterpipe daily. Most of the students (81.0%) said that they smoke less than weekly. Of the waterpipe users 77.6% smoke waterpipe in cafés. The majority of the waterpipe users (91.0%) did not believe they are ‘hooked’ or dependent on the waterpipe.

A total of 91.4% smoke with their friends, and 86.7% share their waterpipe with friends. Of the waterpipe users 21.4% thought of stopping smoking, whereas 62.4% did not.

The perceptions of the smoker and non-smoker students about comparison of harmful effects of waterpipe and cigarette are given in Table IV.

**Table IV.** Perception of the smoker and non-smoker students about comparison of harmful effects of waterpipe and cigarette.

		Waterpipe smoking status							
		Smoker		Non-smoker		Total			
Harmful effects of the waterpipe smoking	Comparison with the cigarette	*n*	%	*n*	%	*n*	%	Chi-square	*P*
Addictivity	Similar	12	5.7	41	9.4	53	8.2	69.71	<0.001
	Less than cigarette	137	65.2	135	31.0	272	42.2		
	More than cigarette	31	14.8	105	24.1	136	21.1		
	Undecided	30	14.3	154	35.4	184	28.5		
Health damage for the smokers	Similar	11	5.2	38	8.7	49	7.6	30.15	<0.001
	Less than cigarette	49	23.3	63	14.5	112	17.4		
	More than cigarette	128	61.0	216	49.7	344	53.3		
	Undecided	22	10.5	118	27.1	140	21.7		
Harmful effect for the other people	Similar	25	11.9	49	11.3	74	11.5	25.92	<0.001
	Less than cigarette	60	28.6	102	23.4	162	25.1		
	More than cigarette	86	41.0	120	27.6	206	31.9		
	Undecided	39	18.6	164	37.7	203	31.5		
Total		210	100.0	435	100.0	645	100.0		

Most of the students thought that waterpipe smoking is less addictive than cigarette smoking. Of the waterpipe users 61% stated that its health damage for the smokers and 41% stated that its harmful effect for other people are greater than cigarettes. These percentages were low among non-smokers of waterpipe (*P* < 0.05). Approximately half of the waterpipe non-users were undecided about the health damage for the smokers, and one-third of them were undecided about the harmful effect for other people.

## Discussion

Almost half of the students in the study group had tried smoking waterpipe at least once, and it was established that approximately one-third still smoked. These findings show that waterpipe smoking is substantially wide-spread among university students.

In a study performed in England among university students, the rate of steady waterpipe smokers was 2.8%; this rate was 19% among waterpipe users in the USA (8,9). In a study performed in Syria the rates of waterpipe smoking were 25.5% among male students, 4.9% among female students, and the rates of daily waterpipe use were 1.8% among male students. In another study performed in Syria, the rates of daily waterpipe use among café customers were 24% (10,11). In East Mediterranean countries, waterpipe is second to cigarette smoking among the types of steady tobacco consumption. The social acceptance of smoking waterpipe, especially the fact that cigarette smoking is seen as shameful for girls whereas waterpipe smoking is not, is also a factor in the steady increase of the smoking rate (5,12). Also it can be seen as a reason for the increase in waterpipe cafés. The increase in waterpipe use in the last years and especially among young people in the East Mediterranean countries is a known fact (2,6).

In the multi-centre study Global Youth and Tobacco Investigation, in its Lebanon part, it was found that among Lebanese youth the cigarette smoking rate had decreased between 2001 and 2005, whereas use of other tobacco methods had increased (13).

In Turkey, which could be thought of as a bridge between the East Mediterranean countries and Europe, and in which the waterpipe is widely used, waterpipe use is expected to be at a lower rate than in the East Mediterranean countries. But on the contrary, in our study we found that the casual use of waterpipe among girls and boys is not low at all.

Although waterpipe use is common among female students, it has been established that the possibility of using waterpipe among male students is twice as high. In a study performed in adolescents in Lebanon, it was found that cigarette smoking alone or together with waterpipe use is much higher in males than in females (5). The study result of the Lebanon 2005 Global Youth Tobacco Investigation has shown that smoking and the use of tobacco other than cigarettes is higher in males than in females (13). However, differing from European countries, in East Mediterranean countries waterpipe smoking is socially accepted, therefore waterpipe use is common in girls living in these countries.

In England, in a study performed with students from the British University, it was found that experiencing waterpipe and its steady use was higher in males (8). In different studies performed in Lebanon in 2001 and in Syria in 2003, in university students, similar results were obtained. In studies performed in Iran and the United States, waterpipe use was found to be higher in boys than in girls (4,12,14).

There are studies that show that, related to the cultural differences between societies, the presence of someone using tobacco products in the family increases the tendency of cigarette smoking in boys and waterpipe smoking in girls. In East Mediterranean countries, waterpipe has been used traditionally for centuries, and among women it is accepted as a less shameful event compared to cigarette smoking. Therefore, waterpipe use is more common in women compared to cigarette smoking. Waterpipe smoking is considered natural among parents, and some parents even smoke it together with their children (5,13,14).

Although waterpipe use was slightly higher in the third-grade students of the faculties, the difference between classes was not significant. This can be explained by the fact that students with a tendency for waterpipe smoking usually do it before university or in the first grade. As a matter of fact the mean age for starting smoking waterpipe was found to be 17. On the other hand, there are studies showing that the rate of smoking waterpipe increased in the higher classes (8,10).

It is known that smoking cigarettes serves as a pioneer for waterpipe smoking, and vice versa (12). In our study we found that the prevalence of waterpipe smoking was nine times greater in students with the habit of cigarette smoking. A similar study, showing that cigarette smoking has an effect upon trying and steadily smoking waterpipe, was done in a British University (8).

In Syria, in a study in which university students and regular café customers were evaluated, the prevalence of waterpipe smokers was found to be higher in cigarette smoking individuals (11). Again in Syria, in a study performed with medical faculty students, similar to our study, waterpipe smoking was found to be ten times more frequent in cigarette smokers compared to non-smokers (15).

The presence of a family member or friend that smokes waterpipe increases substantially the possibility of the student smoking waterpipe as well. In a study performed in the USA, it was found that the presence of a family member smoking waterpipe increases the smoking in other individuals by 6.5 times (12). In our study this value was found to be approximately five times. On the other hand, the presence of a friend smoking waterpipe increases the prevalence by three times. There are many studies showing the important effect of family members and friends upon waterpipe use (1,11–14,16,17).

Most of the students smoke waterpipe less than once a week, and the preferred places to smoke are cafés. Only one student stated that he smoked every day. In a study performed in Ankara, it was found that the preferred smoking sequence was once in a while and not daily (7).

A total of 91% of the waterpipe users did not see themselves as addicted. These findings are similar to the results of two different studies performed in the United States of America (USA). In these studies and in ours, the fact that waterpipe was smoked once in a while by the majority is remarkable. Not seeing themselves as addicts, and the feeling that they can quit whenever they want to, is a common perception among waterpipe users (9,17).

Most of the students were smoking waterpipes with their friends, and collective use was common. In a study from Syria, in which students and café customers were evaluated, it was established that people preferred to smoke with their friends. In the same study, 96.5% of the students and 43.8% of the café customers stated that they smoked the waterpipe collectively (11).

Only 21% of the students in the study group were considering quitting smoking waterpipe. In two different studies performed in the USA (12.2%) and in Turkey (11.0%), the rate of considering quitting was similarly low (7,18). On the other hand in a study performed in Egypt this rate was found to be over 50% (16). The fact that the intensity of waterpipe smoking is low in Turkey and USA, the perception of it as a means of entertainment among friends, and the rejection of its addictive features may all be factors affecting the thoughts of individuals regarding quitting.

The majority of the students in our study who did not smoke waterpipe had no clue about the harmful effects of waterpipe use. The perception that waterpipe use was less addictive compared to cigarette smoking among students smoking waterpipe was significantly higher than in the students not smoking. On the other hand, the fact that most of the students smoking waterpipe thought that it was more harmful to themselves and to the environment compared to cigarette smoking and that this rate was higher than in non-smokers was an interesting finding. This may be due to the fact that the smokers observed more effectively the harmful effects of waterpipe smoking on themselves and on the environment, compared to the non-smokers. In two studies from Syria, performed in university students and café customers, the rate of considering waterpipe smoking as more harmful than cigarette smoking was also high (10,11). On the other hand, there are some studies showing that cigarette smoking is considered to be more harmful compared to waterpipe smoking (8,9,16).

## Conclusion

It was concluded that approximately one-third of the university students smoke waterpipe. Waterpipe smoking was found to be more prevalent among the male and cigarette-smoker students.

The increase observed in waterpipe smoking in the last years poses a danger to the young population. Care should be taken in realizing and taking precautions about these kinds of dangerous situations, and awareness should be raised among young people regarding its dangers.

In order to achieve success in the struggle against waterpipe use among young boys and girls, we should focus on the wrong perceptions about waterpipes, such as its being less harmful and more glorious, and its use, especially among youth, should be prevented. Also, support should be given for healthy activities in order to provide some help with changing behavioural patterns.
